# Ca^2+^-Dependent Protein Kinase 6 Enhances KAT2 Shaker Channel Activity in *Arabidopsis thaliana*

**DOI:** 10.3390/ijms22041596

**Published:** 2021-02-05

**Authors:** Elsa Ronzier, Claire Corratgé-Faillie, Frédéric Sanchez, Christian Brière, Tou Cheu Xiong

**Affiliations:** 1 BPMP, University Montpellier, CNRS, INRAE, Institut Agro, 34060 Montpellier, France; elsa.ronzier@gmail.com (E.R.); claire.corratge-faillie@cnrs.fr (C.C.-F.); sanchezf@obs-banyuls.fr (F.S.); 2 BIOM 7232, Avenue Pierre Fabre, 66650 Banyuls-Sur-Mer, France; 3 Laboratoire de Recherche en Sciences Végétales, UMR CNRS/UPS 5546, 24 chemin de Borde Rouge, 31326 Castanet-Tolosan, France; christian.briere@sfr.fr

**Keywords:** *Arabidopsis thaliana*, shaker channel, CPK, calcium signaling, post-translational regulation

## Abstract

Post-translational regulations of Shaker-like voltage-gated K^+^ channels were reported to be essential for rapid responses to environmental stresses in plants. In particular, it has been shown that calcium-dependent protein kinases (CPKs) regulate Shaker channels in plants. Here, the focus was on KAT2, a Shaker channel cloned in the model plant *Arabidopsis thaliana*, where is it expressed namely in the vascular tissues of leaves. After co-expression of KAT2 with AtCPK6 in *Xenopus* *laevis* oocytes, voltage-clamp recordings demonstrated that AtCPK6 stimulates the activity of KAT2 in a calcium-dependent manner. A physical interaction between these two proteins has also been shown by Förster resonance energy transfer by fluorescence lifetime imaging (FRET-FLIM). Peptide array assays support that AtCPK6 phosphorylates KAT2 at several positions, also in a calcium-dependent manner. Finally, K^+^ fluorescence imaging *in planta* suggests that K^+^ distribution is impaired in *kat2* knock-out mutant leaves. We propose that the AtCPK6/KAT2 couple plays a role in the homeostasis of K^+^ distribution in leaves.

## 1. Introduction

Plant Shaker channels are animal voltage-gated K^+^ channel homologs resulting from the assembly of four subunits and ubiquitously expressed in plants. In the plant model *Arabidopsis thaliana*, the Shaker channel subunits are encoded by nine genes: *GORK*, *SKOR*, *AKT5*, *AKT6*, *AKT1*, *AKT2*, *AtKC1*, *KAT1,* and *KAT2* [1,2,3,4]. Potassium (K^+^) channels KAT2 and AKT2 are expressed in plant vascular tissues such as phloem [5,6,7]. While KAT2 expression is restricted to leaves, AKT2 is expressed in the roots and leaves phloem. AKT2 is involved in K^+^ homeostasis in phloem and has been shown to be regulated by a Calcineurin-B-*like* (CBL)/CBL-interacting protein kinase (CIPK) couple belonging to a large family of complexes known to be important relays in plant calcium (Ca^2+^) signaling [8,9]. Moreover, heteromerization of KAT2 and AKT2 has been shown, potentially increasing the diversity of properties of the resulting Shaker channels [5]. Both KAT2 and AKT2 are likely to play a role in potassium long distance transport and distribution in plant and as a consequence, both are involved in the maintenance of K^+^ homeostasis in the whole plant [10,11]. Moreover, the expression of KAT2 and AKT2 have been previously found in vascular tissues of *Arabidopsis thaliana* [6,12,13]. The localization of these ion channels and their fine post-translational modification by second messengers are keys for an adequate regulation and a rapid adaptation of plants to stresses.

Upon stresses, Ca^2+^ signals are observed and characterized by their intensity, duration, frequency, and localization [14,15]. Decoding of these Ca^2+^ signals by a network of Ca^2+^-sensing proteins such as calmodulin (CaM), Calcineurin B-*Like* proteins (CBLs), or Ca^2+^ dependent protein kinases (CPKs or also known as CDPKs) lead to adapted plant response [11,16,17]. 

CPKs that are unique in the plant kingdom are serine/threonine kinases that bear a CaM domain and allow Ca^2+^-signal transduction through protein phosphorylation events. CPKs are involved in plant development such as germination, plant growth, and flowering [18,19]. CPKs also play roles in plant responses to biotic and abiotic stresses [20,21]. For example, StCDPK7, OsCPK12, OsCPK4, and AtCPK6, respectively, expressed in potato (*Solanum tuberosum*, St), rice (*Oryza sativa*, Os), and *Arabidopsis thaliana* (At) are involved in disease resistance [22,23,24,25]. Regarding abiotic stress, it has been shown that OsCPK6, OsCPK13, OsCPK17, OsCPK24, and OsCPK25 are involved in cold stress tolerance [26,27]. Interestingly, AtCPK1, AtCPK3, AtCPK6, and AtCPK23 play a role in the sodium ion tolerance and AtCPK6, AtCPK21, and McCPK1 from Iceplant (*Mesembryanthemum crystallinum*, Mc) are involved in osmotic stress responses [28,29,30]. 

CPKs can enhance or inhibit channel activities and regulate ion transport across the plasma membrane [31,32,33,34,35]. In *Vicia faba* (Vf), it has been found that VfCPK phosphorylates and inhibits KAT1, a K^+^ Shaker channel of guard cells, the two cells surrounding stomata that allow a tight control of plant water loss [36]. Similar work performed with CPK from soybean showed an inhibition of KAT1 activity in oocytes [37]. In *Arabidopsis thaliana*, little is known about the targets of the 34 members of the CPK family. AtCPK6, AtCPK21, and AtCPK23 could activate SLAC1 (Slow Anion Channel-associated 1) in guard cells [32,38]. AtCPK13 phosphorylates and inhibits KAT1 and KAT2 and leads to stomatal closure [35]. AtCPK33 was shown to stimulate GORK channels [31]. Shaker channels are therefore targeted and regulated by CPKs.

Precisely, post-translational modifications of Shaker channels could participate to fine tune K^+^ fluxes in order to adapt plants for growth and stress responses. It has been demonstrated that the overexpression of AtCPK6 confers salt and drought stress tolerance to plants [28]. In order to understand this mechanism, we investigated the role of AtCPK6 on Shaker channels. Functional screening by heterologous expression in *Xenopus laevis* oocytes shows that AtCPK6 stimulates inward and outward K^+^ currents [31]. Here, we show that *AtCPK6* is widely expressed in the whole plant including vascular tissues. Both *AtCPK6* and *KAT2* are expressed in vascular tissues, then we hypothesized that KAT2 could be a target for AtCPK6. To support this, we used an electrophysiological approach to show that AtCPK6 enhanced KAT2 channel activity only in the presence of free Ca^2+^. We have also demonstrated that AtCPK6 interacts with KAT2 *in planta* and using peptide arrays, we have found that recombinant AtCPK6 kinase phosphorylates KAT2 peptides in vitro in a Ca^2+^ dependent manner. Finally, using fluorescence dye to image K^+^, we observed that *kat2* knock-out plants present a K^+^ distribution defect in leaves. Taking all these results together, we propose that AtCPK6 activates KAT2 in the leaves’ vascular tissues in the presence of Ca^2+^ to allow for a rapid change of K^+^ distribution during environmental stresses.

## 2. Results

### 2.1. AtCPK6 Is Highly Expressed in Vascular Tissues

**Figure 1:**
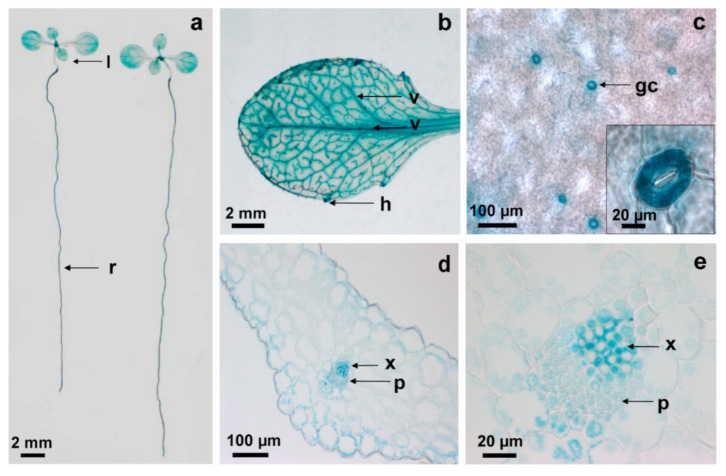
*AtCPK6* expression pattern in *Arabidopsis thaliana.* Representative pictures of (**a**) p*AtCPK6*-GUS *Arabidopsis thaliana* whole plant with leaves (l) and roots (r); (**b**) leaf with veins (v) and hydathodes (h) after GUS staining; and (**c**) zoom from a leaf showing guard cells (gc), and (**d**,**e**) sections of root showing xylem (x) and phloem (p).

In order to localize *AtCPK6* expression *in planta*, we generated a transgenic *Arabidopsis thaliana* line expressing the *AtCPK6* promoter fused with β-glucuronidase gene reporter (GUS). *AtCPK6* promoter activity was revealed by high GUS staining in roots and leaves (Figure 1a,b) in guard cells (Figure 1c) and interestingly in the vascular tissues within phloem and xylem (Figure 1b,d,e). We then investigated whether AtCPK6 could regulate the activity of KAT2 and AKT2, which have both been previously shown to be expressed in plant vascular tissues [6,12,13].

### 2.2. AtCPK6 Enhances Specifically KAT2 Channel Activity in Oocytes

**Figure 2:**
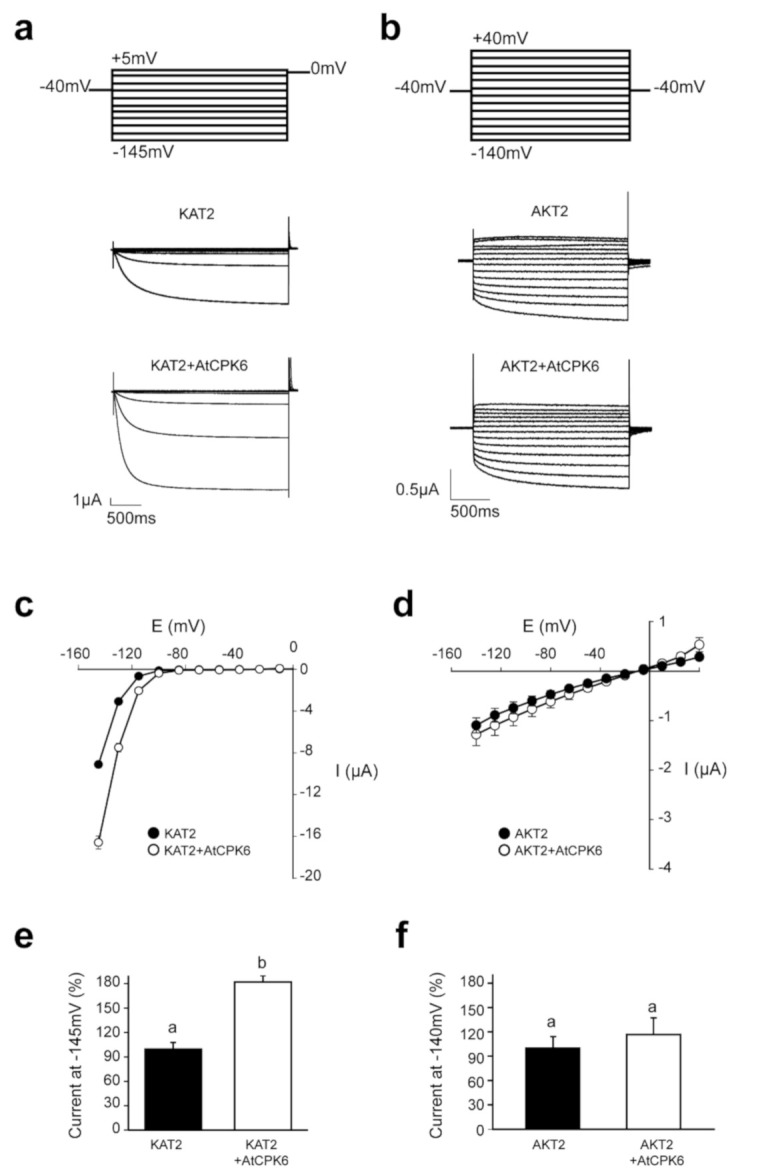
AtCPK6 specifically activates KAT2 Shaker channel in *Xenopus laevis oocytes*. Representative current recordings obtained by TEVCs (**a**) with voltage step values ranging from +5 mV to −145 mV in *Xenopus laevis* oocytes expressing KAT2 or KAT2 + AtCPK6 or (**b**) with voltage step values ranging from +40 mV to −140 mV in oocytes expressing AKT2 or AKT2 + AtCPK6. Average current–voltage relationships obtained from these TEVC recordings with *Xenopus laevis* oocytes expressing (**c**) KAT2 (black circles, *n* = 19 oocytes, from 3 independent experiments) or KAT2 + AtCPK6 (white circles, *n* = 17 oocytes, from 3 independent experiments) and (**d**) AKT2 (black circles, *n* = 13 oocytes, from three independent experiments), or AKT2 + AtCPK6 (white circles, *n* = 12 oocytes, from three independent experiments). Average value of current (**e**) at −145mV in oocytes expressing KAT2 or KAT2 + AtCPK6 or (**f**) at −140mV in oocytes expressing AKT2 or AKT2 + AtCPK6. One-way ANOVA tests with post-hoc Tukey were performed. Different letters indicated significance for *p* ≤ 0.001.

KAT2 and AKT2 activities were measured by two electrode voltage-clamp (TEVC) in *Xenopus laevis* oocytes expressing KAT2 or AKT2 subunits with or without AtCPK6. Typical recordings and average I/V curves obtained are presented in Figure 2a–d. A significant two-fold increase of KAT2 currents upon co-expression with AtCPK6 was observed at hyperpolarized potentials (Figure 2c,e) whereas AKT2 currents did not show any significant change upon co-expression with AtCPK6 (Figure 2d,f). This suggests that KAT2 is selectively targeted by AtCPK6 in a heterologous expression system such as *Xenopus laevis* oocytes.

### 2.3. KAT2 Is Stimulated by AtCPK6 in Ca^2+^-Dependent Manner

**Figure 3:**
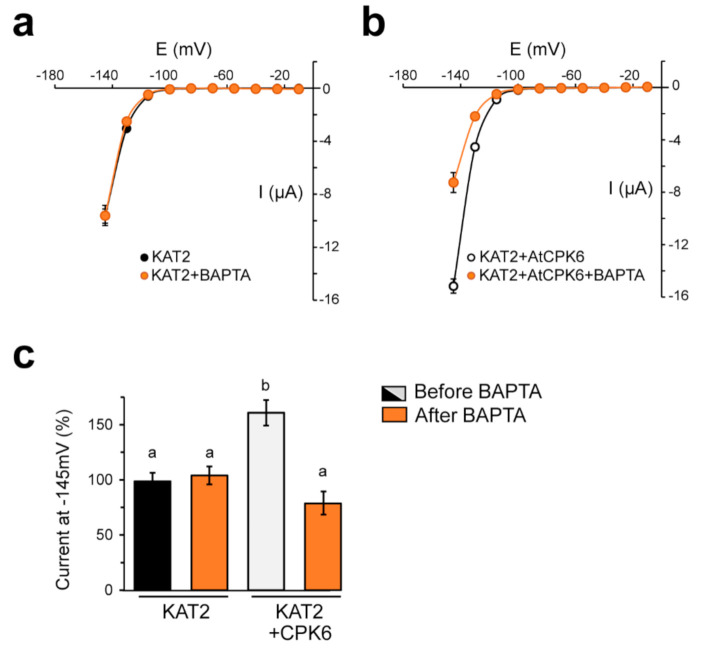
AtCPK6 stimulates KAT2 Shaker channel in *Xenopus laevis oocytes* in calcium dependent manner. Current–voltage relationships obtained from TEVC recordings of (**a**) oocytes expressing KAT2 before (*n* = 6 oocytes from two independent experiments) and after BAPTA injection (*n* = 5 oocytes from two independent experiments) and (**b**) of oocytes expressing KAT2 + AtCPK6 before (*n* = 6 oocytes from 2 independent experiments) and after BAPTA injection (*n* = 5 oocytes from two independent experiments). (**c**) Average value of current recorded at −145 mV in oocytes expressing KAT2 or KAT2 + AtCPK6 before and after BAPTA injection. One-way ANOVA tests with post-hoc Tukey were performed. Different letters indicate significance for *p* ≤ 0.001.

Most of the CPKs displayed different kinase activities depending on their affinity to free Ca^2+^ [39]. AtCPK6 is a strictly Ca^2+^ dependent kinase [40] (Figure S1c). In order to confirm that the increase of KAT2 activity was due to Ca^2+^ activation of AtCPK6, a calcium chelator (BAPTA) was injected in the *Xenopus laevis* oocyte expressing either KAT2 alone or KAT2 with AtCPK6. BAPTA injection had no effect on KAT2 activity when expressed alone (Figure 3a). However, injection of BAPTA in oocytes expressing KAT2 + AtCPK6 had a significant effect by abolishing AtCPK6 stimulation of KAT2 currents (Figure 3b). In the presence of BAPTA, KAT2 currents were similar in oocytes expressing KAT2 with AtCPK6 and in oocytes expressing KAT2 alone (Figure 3c), suggesting that the increase of K^+^ inward currents ascribed to AtCPK6 are Ca^2+^-dependent.

### 2.4. AtCPK6 Physically Interacts with the KAT2 Subunit Channel in Planta

**Figure 4:**
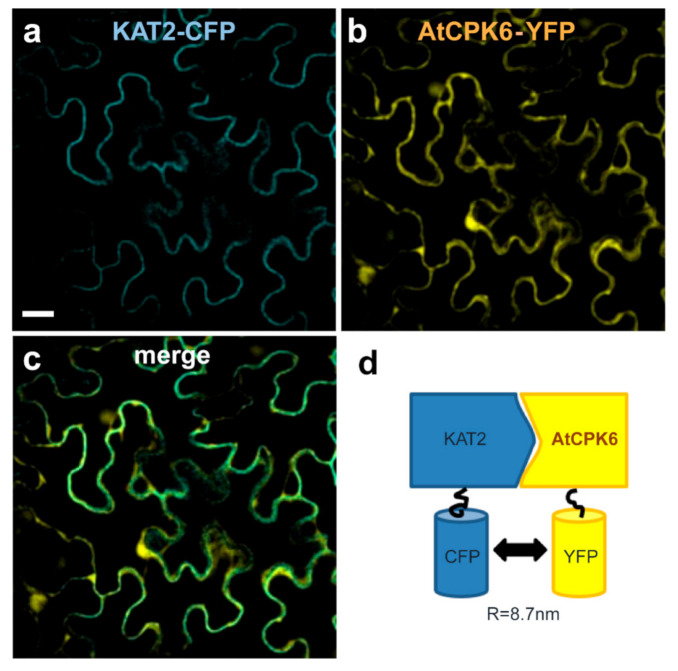
AtCPK6 and KAT2 colocalize and interact in plant cells. Representative confocal fluorescence microscopy pictures of *N. benthamiana* epidermal leaf cells co-expressing KAT2-CFP and AtCPK6-YFP showing (**a**) KAT2-CFP, (**b**) AtCPK6-YFP, and (**c**) merge. *In planta* schematic representation of KAT2-CFP and AtCPK6-YFP interaction obtained with the FRET-FLIM experiment (**d**) in *N. benthamiana* epidermal leaf cells displaying a distance of 8.7 nm (see Table 1). Results were obtained in three independent experiments. The distance (R) between CFP and YFP was calculated by Foster’s equation: $E\;=\;\frac{1}{1+{\left(\frac{R}{R_{0}}\right)}^{6}}$ with $R_{0}\;=\;58\;Å$ for CFP and YFP [41].

The stimulation of KAT2 currents by AtCPK6 in the presence of Ca^2+^ suggests that AtCPK6 might interact with KAT2. The physical interaction between the two partners, AtCPK6 and KAT2, was therefore investigated *in planta* with a Förster resonance energy transfer by fluorescence lifetime imaging (FRET-FLIM) experiment using *N. benthamiana.* KAT2 and AtCPK6 were fused with CFP and YFP, respectively. KAT2-CFP was expressed alone or co-expressed with AtCPK6-YFP by *agrobacterium* infiltration in *N. benthamiana* leaves. Using confocal microscopy, we found that KAT2-CFP and AtCPK6-YFP co-localized at the plasma membrane (Figure 4a–c). A physical interaction between KAT2-CFP and AtCPK6-YFP was then investigated by detecting FRET events between the donor (CFP) and the acceptor (YFP). Changes of CFP fluorescence lifetime were measured in two conditions: KAT2-CFP alone or KAT2-CFP with AtCPK6-YFP. Results show that the CFP lifetime was significantly reduced in the presence of AtCPK6-YFP compared to KAT2-CFP alone (Figure 4d, Table 1).

### 2.5. AtCPK6 Is Able to Phosphorylate KAT2 Peptides in the Presence of Free Ca^2+^

**Figure 5:**
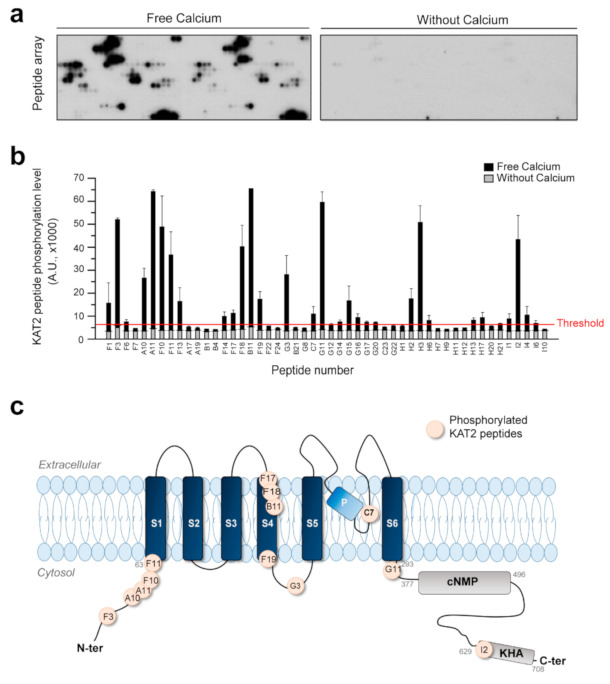
Putative phosphorylation sites of KAT2 Shaker channel targeted by AtCPK6. Representative autoradiographs of peptide array targeting all the serines and threonines of KAT2 (**a**) after incubation with recombinant AtCPK6 in the presence (free calcium, left) or in the absence of Ca^2+^ (without calcium, right). Stained black spots correspond to phosphorylated peptides. Quantification of KAT2 peptide phosphorylation by AtCPK6 (**b**) in the presence (black bars) or in the absence (grey bars) of Ca^2+^. Results were obtained from one peptide array containing peptide in duplicate. Schematic drawing of the secondary structure of the KAT2 Shaker channel subunit (**c**), highlighting the putative sites phosphorylated by AtCPK6 (pink dots) as deduced from the peptide array. S1 to S6, P, cNMP, and KHA stand for transmembrane segments 1–6, pore domain, putative cyclic nucleotide-binding domain, and domain rich in hydrophobic and acidic residues, respectively.

AtCPK6 is a serine/threonine kinase phosphorylating protein targets and substrates. As a consequence, we produced and purified AtCPK6 recombinant protein in order to study Shaker channel phosphorylation events in vitro. Purification of recombinant AtCPK6 as well as its kinase activity were validated by biochemistry with sodium dodecyl sulfate polyacrylamide gel electrophoresis (SDS-PAGE) and in vitro kinase assays (Supplementary Figure S1a,b). Recombinant AtCPK6 strongly phosphorylates syntide-II, a well-known substrate of AtCPKs (Supplementary Figure S1b). With this fully functional recombinant AtCPK6, we studied phosphorylation events in vitro using a peptide array containing a set of Shaker channel peptides (Supplementary Table S1) and phosphorylation positive controls including syntide-II (Supplementary Figure S2). The potential phosphorylation sites (serine and threonine residues) of KAT2 were predicted to generate 52 KAT2 peptides that cover the entire amino acid of the KAT2 sequence. These peptides were synthetized and spotted on a peptide array (Supplementary Table S1). Therefore, the 55 serines and 40 threonines of KAT2 were present on the peptide array. A similar approach was done for the AKT2 and 60 peptides designed to cover the AKT2 sequence. In vitro phosphorylation of KAT2 and AKT2 peptides was then performed with the recombinant AtCPK6 protein with 830 µM estimated free Ca^2+^ or without Ca^2+^ (Figure 5a). Radiolabeling of peptides targeted by AtCPK6 in the presence or absence of free Ca^2+^ is shown in Figure 5a. In the presence of Ca^2+^, several peptides were phosphorylated by AtCPK6 whereas in the absence of Ca^2+^, few peptides with very weak signals were observed (Figure 5a). Quantification of the phosphorylated peptide signals of KAT2 and AKT2 is shown in Figure 5b and Supplementary Table S1. It seems that AtCPK6 phosphorylates 10 AKT2 peptides, specifically localized in the N-terminal part and in transmembrane domains of AKT2 (Supplementary Figure S3 and Table S2). Regarding KAT2, we found that 19 KAT2 peptides were selectively targeted and phosphorylated by AtCPK6 in the presence of Ca^2+^ (Supplementary Table S2). The 19 peptides contained 22 serines and 15 threonines, which were localized in the N-terminal part, in the transmembrane segment S4, and in the C-terminal part of KAT2 (Figure 5c).

### 2.6. Potassium Distribution Is Perturbed in kat2 Knock-out Plants

**Figure 6:**
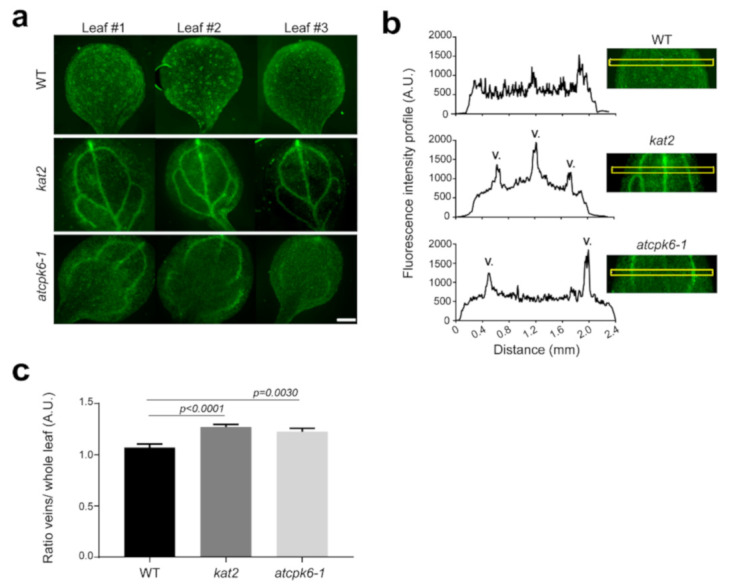
Potassium distribution in leaves. Representative fluorescence pictures (**a**) of seven day old wild-type (WT), *kat2*, and *atcpk6-1* plants after 3 h incubation with 20 µM Asante K^+^ green probe. (**b**) Representative fluorescence intensity profiles (left) of leaf cross sections for WT, *kat2*, and *atcpk6-1*, (**c**) averages of K^+^ fluorescence ratios of veins/whole leaf (*n* = 22 leaves for WT; *n* = 23 leaves for *kat2*; *n* = 21 leaves for *atcpk6-1* from three independent experiments). Mann–Whitney test, two-tailed was performed. Scale bar = 500 µm.

In order to study the physiological role of KAT2 Shaker channel *in planta*, we confirmed that *KAT2* is expressed in the emerging leaves’ vasculatures of *Arabidopsis thaliana* (Supplementary Figure S4) as previously shown by Pilot et al. and Philippar et al. [12,13]. We then used a K^+^ fluorescent probe to study potassium distribution in wild-type, *kat2* and *atcpk6-1* mutant plant leaves (Figure 6a). We observed a drastic change in the K^+^ distribution in the *kat2* mutant with a strong fluorescent K^+^ signal mainly localized in veins (primary, secondary, and tertiary veins) while K^+^ is widely localized in the entire leaf of wild-type plants. Fluorescence intensity profiles of the leaf cross sections showed that K^+^ distribution is strongly impaired for *kat2*, moderately impaired for *atcpk6-1* mutant, and not at all for wild-type leaves (Figure 6b). We quantified the K^+^ fluorescence ratio between veins and the whole leaf (Figure 6c). We found a higher ratio for *kat2* in comparison with the wild-type and *atcpk6-1* plants. Interestingly, we also noticed a strong signal in the entire leaf of *atcpk6-1* plants including secondary and tertiary veins with often a lack of signal in the primary vein explaining a lower ratio primary vein/whole leaf. Taken together, these results indicate that the loss of KAT2 and even AtCPK6 leads to an accumulation of K^+^ in veins instead of an equal distribution through the whole leaf.

## 3. Discussion

KAT2 is expressed in stomatal guard cell and in leaf vascular tissues [12,13]. Its function was characterized in plant stomata guard cells allowing stomatal opening [12,42]. Our recent work has shown that KAT2 activity inhibition by AtCPK13 leads to stomatal aperture defect [35]. However, the role of KAT2 in leaf vascular tissues remains unclear. Here, our results suggest that KAT2 is involved in K^+^ distribution in plants. The *kat2* mutant plant does not show any phenotype compared to WT in physiological condition. However, fluorescence imaging using Asante K^+^ green dyes showed that *kat2* and *atcpk6-1* leaf veins were enriched in K^+^ compared to wild-type plants. This K^+^ distribution defect has not been previously reported in the *kat2* mutant. Altogether, *KAT2* expression in the phloem and K^+^ distribution defect in the *kat2* mutant suggest that KAT2 could be involved in K^+^ phloem loading. The increase of K^+^ staining in vascular tissues in *kat2* was not expected, but pointed out the fact that K^+^ allocation is impaired from phloem and the surrounding cells. A precise localization with higher magnification will be investigated, but the compensatory effect by other potassium channels such as AKT2 was not excluded to explain this increase of K^+^ in leaf veins.

K^+^ staining in the *atcpk6-1* mutant background shows that K^+^ is mainly concentrated in secondary veins compared to the *kat2* mutant showing a strong K^+^ staining in both primary and secondary veins. *AtCPK6* is widely expressed in the plant. GUS staining shows that *AtCPK6* promoter is active in both phloem and xylem poles. Despite the fact that AtCPK6 might have other targets in the phloem, the lack of KAT2 stimulation by AtCPK6 could explain the misallocation of the K^+^ in leaves.

We showed that AtCPK6 stimulates KAT2 activity in the presence of Ca^2+^ in oocytes (Figure 2 and Figure 3). Notably, the basal level of free cytosolic Ca^2+^ was enough to activate AtCPK6 and then stimulated KAT2 activity. In particular, we found that 0.1 µM of Ca^2+^ was sufficient to increase AtCPK6 kinase activity by a factor of 1.8 and 2 µM was sufficient to trigger the maximum activity of AtCPK6 (Supplementary Figure S1c). In plants, the cytosolic free Ca^2+^ level was reported to be around 0.1 µM and should also be sufficient to stimulate KAT2. Upon environmental stresses (e.g., salt stress), increases of free Ca^2+^ level located in the veins on leaves have been reported [43] and changes of KAT2 activity through AtCPK6 in leaf vascular tissues might participate in long distance signaling from roots to leaves. In this work, we showed that KAT2 phosphorylation by AtCPK6 is strictly dependent of Ca^2+^. Indeed, we identified potential phosphorylation sites for KAT2 by AtCPK6 using peptide array assays (Figure 5). AtCPK6 was able to selectively phosphorylate 19 peptides among the 52 KAT2 peptides tested. In a previous study, we showed that 11 KAT2 peptides are targeted by AtCPK13, a non-calcium dependent kinase [35]. Among these 11 peptides, some are shared with the ones described in this new study. Therefore, only eight peptides could be phosphorylated upon Ca^2+^ signal. It seems then that there are multiple phosphorylation sites that are potentially involved in the down- or upregulation of KAT2 activity. We found that the N-terminal residues of AKT2 are phosphorylated by AtCPK6 (Supplementary Figure S3), but this does not affect AKT2 activity (Figure 1). Regarding these results, the phosphorylated N-terminal residues of the Shaker channels did not seem to be involved in channel activity regulation. Next, we proposed that KAT2 positive regulation by AtCPK6 is potentially due to the pore region and/or C-terminal residue phosphorylation. In the near future, we will use these results as guidance to specifically target serine/threonine residues involved in KAT2 upregulation.

Finally, it has been shown that Ca^2+^ signals induce *AtCPK6* expression [44] and stimulate its kinase activity (Figure 3 and Figure 5). Moreover, Ca^2+^ waves have been shown in plant vascular tissues under salt stress [43]. Taking the data available in the literature and our new results presented here together, we hypothesize that the upregulation of AtCPK6 expression and activity by Ca^2+^ signals in *Arabidopsis* leaves could contribute to the early step of plant adaptation. Here, we propose that AtCPK6, activated by Ca^2+^, could stimulate KAT2 activity in leaf vascular tissues to maintain K^+^ homeostasis. Further investigations will be made in this direction to understand the role of post-translational regulation of KAT2 in salt stress.

## 4. Materials and Methods

### 4.1. Wild-Type and Mutant Plants

*Arabidopsis thaliana* plant ecotype Colombia (Col-0) was used as the wild-type in the experiments. The T-DNA knock-out mutant plants *kat2* (Salk_025933/N525933) and *atcpk6-1* (salk_093308/N593308) were provided by the Nottingham Arabidopsis Stock Center (NASC).

### 4.2. Molecular Biology

Full-length complementary coding regions (cDNAs) of *KAT2*, *AKT2*, *AtCPK6*, and *AtCPK6* promoter (2 kbp upstream of start codon) were introduced into a Gateway *pDONR207* vector by recombination, according to the manufacturer’s instructions (BP cloning; Invitrogen). Primers used for BP cloning are listed in Supplementary Table S3. Gateway LR recombinations were then performed between entry clones and several destination vectors: *pGEMGWC* for heterologous expression in *Xenopus laevis* oocytes [35], *pDEST15* for recombinant protein production in Rosetta strain [35]; and *pEarleyGate101* and *102* for CFP and YFP fusion, respectively [45].

### 4.3. Transgenic Plants

The promoter of *AtCPK6* (2 kb) was cloned into pGWB533 to generate pro*CPK6* fused with the β-glucorosidase reporter enzyme [46]. The resulting plasmid was introduced *A. tumefaciens* (GV3101:pMP90) for *Arabidopsis thaliana* transformation using the floral dip method [47]. T2 homozygous transgenic plants were selected by hygromycin resistance (50 µg·mL^−1^) in one-half-strength Murashige and Skoog medium (MS) supplemented with sucrose (1%, *w*/*v*).

### 4.4. GUS Staining

Plantlets were incubated in a pre-fix solution (50 mm NaH_2_PO_4_, 1.5% [*v*/*v*] formaldehyde, and 0.05% [*v*/*v*] Triton X-100, pH 7) and then in a GUS-fix solution (50 mM NaH_2_PO_4_, 0.5 mM ferricyanide, 0.5 mM ferrocyanide, 0.05% [*v*/*v*] Triton X100, and 1 mM 5-bromo-4-chloro-3-indolyl-β-d-glucuronic acid, pH 7) overnight at 37 °C. On the day after, plantlets were washed in successive baths of 50%, 70%, 90%, and 100% (*v*/*v*) of ethanol. Observations were made using a stereomicroscope Olympus and pictures were acquired with a Color-View II camera (Olympus).

### 4.5. Two Electrodes Voltage–Clamp (TEVC)

Oocyte preparation, injection, and two electrode voltage–clamp (TEVC) measurements were performed as described [31,35]. In vitro transcriptions were performed as described in Ronzier et al. (2014) [35]. cRNAs of *KAT2*, *AKT2*, and *AtCPK6* were obtained using the MESSAGE mMACHINE T7 Ultra Kit according to the manufacturer’s instructions (Ambion). Oocytes were injected with either 20 ng of *KAT2* cRNA alone, or co-injected with 20 ng of *KAT2* cRNA + 20 ng of *AtCPK6* cRNA. Similar protocols were used for *AKT2* and *AtCPK6*. After cRNA injection, oocytes were incubated for 48 h at 20 °C in a solution containing 96 mM NaCl, 2 mM KCl, 1.8 mM CaCl_2_, 2 mM MgCl_2_, 2.5 mM Na-pyruvate, 5 mM HEPES-NaOH (pH 7.5), and 50 µg mL^−1^ gentamycin. KAT2 or AKT2 currents were recorded using the TEVC technique with oocytes bathed in 10 mM KCl, 90 mM NaCl, 1 mM MgCl_2_, 1 mM CaCl_2_, and 10 mM HEPES-NaOH (pH 7.5). The voltage protocol was composed of 3 s voltage pulses ranging from +5 mV to −145 mV (for KAT2) or from +40 mV to −140 mV (for AKT2) with −15 mV decrements starting from a holding potential of −40 mV. For the BAPTA experiments, currents were first recorded from oocytes expressing KAT2, then these oocytes were injected with BAPTA. After 5 min incubation, KAT2 currents were then recorded again following the same protocol of TEVC.

### 4.6. Fluorescence Lifetime Imaging (FLIM)

FLIM experiments were performed as described in Ronzier et al. (2014) [35]. Here, leaves of *N. benthamiana* were infiltrated with *Agrobacterium tumefaciens* harboring the *pEarleyGate* plasmids: p101-*AtCPK6* and p102-*KAT2*. FRET-FLIM results were calculated from Forster’s equation with comparisons between the control (KAT2-CFP alone) and co-expressed (KAT2-CFP + AtCPK6-YFP).

### 4.7. K^+^ Fluorescence Dye Staining

K^+^ staining was performed respectively with Asante K^+^ green (APG-2, TEFLab). Seven day old seedlings were transferred on ½ MS media. Agar media were removed to avoid contact between the media and cotyledons. Only roots were then in contact with the medium. The staining was performed as described by Wang et al. [48]. Briefly, seedlings were incubated in the buffer with 20 µM dye (5 mM NaCl, 5 mM CaCl_2_, 5 mM MES pH 6.1) for 3 h in the dark at room temperature. The seedlings were washed twice in the buffer before observation under microscopy at 5X magnification with 450–490 nm excitation/500–550 nm emission (Zeiss Axio Observer Z1/7). Pictures were analyzed with ImageJ freeware. The automatic default threshold was used to remove unstained and background signals and segmented line tool was used to quantify the fluorescence of the vein of leaves.

### 4.8. Recombinant Protein Production and Purification

Recombinant AtCPK6 protein has been produced and purified from Rosetta *E. coli* strains expressing pDEST15-*AtCPK6* plasmid. Rosetta *E. coli* were grown in 200 mL of Löwenstein-Jensen TB medium with ampicillin and chloramphenicol at 50 and 30 µg·mL^−1^, respectively. When the optical density at 600 nm reached 0.7 to 0.8, protein production was started with 0.8 mM–1 mM of isopropyl β-d-1-thiogalactopyranoside (IPTG). After 12 h of culture at 18 °C, bacteria were centrifuged and the pellet suspended in phosphate-buffered saline (PBS) at pH 7.3 supplemented with 0.5 mg mL^−1^ lysozyme (Roth) and protease inhibitor cocktails (Roche). Cells were then sonicated, centrifuged at 12,000× *g* at 4 °C for 12 min and the lysate was incubated with 500 µL of beads glutathione sepharose 4B (GE Healthcare) at 4 °C for 2.5 h. Beads were finally washed four times.

### 4.9. In Vitro Phosphorylation

For kinase activity of recombinant AtCPK6: Using a kinase Glow Kit (Promega, Kinase-Glo Luminescent Kinase Assays), 500 ng of recombinant AtCPK6 was incubated with 25 µM of Syntide-2 and 25 µM of ATP in kinase buffer (Hepes, 50 mM, MgCl_2_ 10 mM, DTT 2 mM, EGTA 4 mM, CaCl_2_ 4.86 mM, pH 7.5). After 15 min incubation, 50 µL of Kinase Glo reagent was added. The remaining ATP was determined using spectrophotometer (Victor, Perkin Elmer Life Science).

### 4.10. Peptide Array Phosphorylation

The peptide arrays were designed as described in Ronzier et al. (2014) [35]. For in vitro phosphorylation assays, peptide arrays were incubated 1 h at 25 °C upon slow agitation with 5 µg of recombinant AtCPK6 in 5 mL of a kinase buffer (10 mM MgCl_2_, 2 mM dithiothreitol, 4 mM EGTA, and 50 mM HEPES-NaOH, pH 7.4) without or with 4.86 mM CaCl_2_ (830 µM free Ca^2+^estimated with Maxchelator Freeware) and with 5 µCi of [γ-^32^P] ATP. Peptide arrays were then washed five times using 5 mL of washing solution (150 mM NaCl, 5 mM EDTA, 0.05% [*v*/*v*] Triton X-100, and 50 mM HEPES-NaOH, pH 7.4). Radiolabeling of phosphorylated peptides was revealed by autoradiography. Quantification of phosphorylation level was performed by image analysis using ImageJ freeware.

## 5. Conclusions and Perspective

Here, we identified and characterized AtCPK6 as a positive activator of the KAT2 Shaker channel expressed in phloem. This regulation is Ca^2+^-dependent and might contribute to adapt K^+^ distribution in plant leaves. We propose that KAT2 channels expressed in vascular tissues are involved in potassium loading/distribution from the phloem to the whole leaf in order to maintain homeostasis during stress. Consequently, the activity of KAT2 channels in ionic stress (salt stress) conditions will be studied. The molecular role of KAT2/AtCPK6 will also be defined using an imaging approach on *Arabidopsis thaliana* mutant plants in various environmental stress chambers/conditions.

## Figures and Tables

**Table 1:** FRET-FLIM measurements.

	Fluorescence Lifetime of CFP (ns) ± s.e.	Change in CFP Fluorescence Lifetime (%)	Number of Measures
**KAT2-CFP**	2.23 ± 0.02	--	22
**KAT2-CFP + AtCPK6-YFP**	2.05 ± 0.03	8.1	25

## Data Availability

The data presented in this study are available in Supplementary Material.

## Supplementary Materials

The following are available online at https://www.mdpi.com/1422-0067/22/4/1596/s1. Figure S1: Calcium-dependent activity of AtCPK6 recombinant protein, Figure S2: In vitro phosphorylation of control peptides by AtCPK6, Figure S3: Potential phosphorylation sites of AKT2 channel by AtCPK6, Figure S4: Expression pattern of KAT2 in *Arabidopsis thaliana.* Table S1: List of KAT2 peptides included in the peptide array, Table S2: List of AKT2 peptides included in the peptide array. Table S3: List of primers used in this study to clone genes.
