# Supplementary material for: Ca2+-Dependent Protein Kinase 6 Enhances KAT2 Shaker Channel Activity in Arabidopsis thaliana
Source: Int J Mol Sci. 2021 Feb 5;22(4):1596. doi: 10.3390/ijms22041596 (PMC7914964; doi:10.3390/ijms22041596)
Supplement: Supplementary file 1 [file ijms-22-01596-s001.zip › Supplemental figures_Ronzier et al.docx]

**SUPPLEMENTAL FIGURES**

Supplemental Figure S1


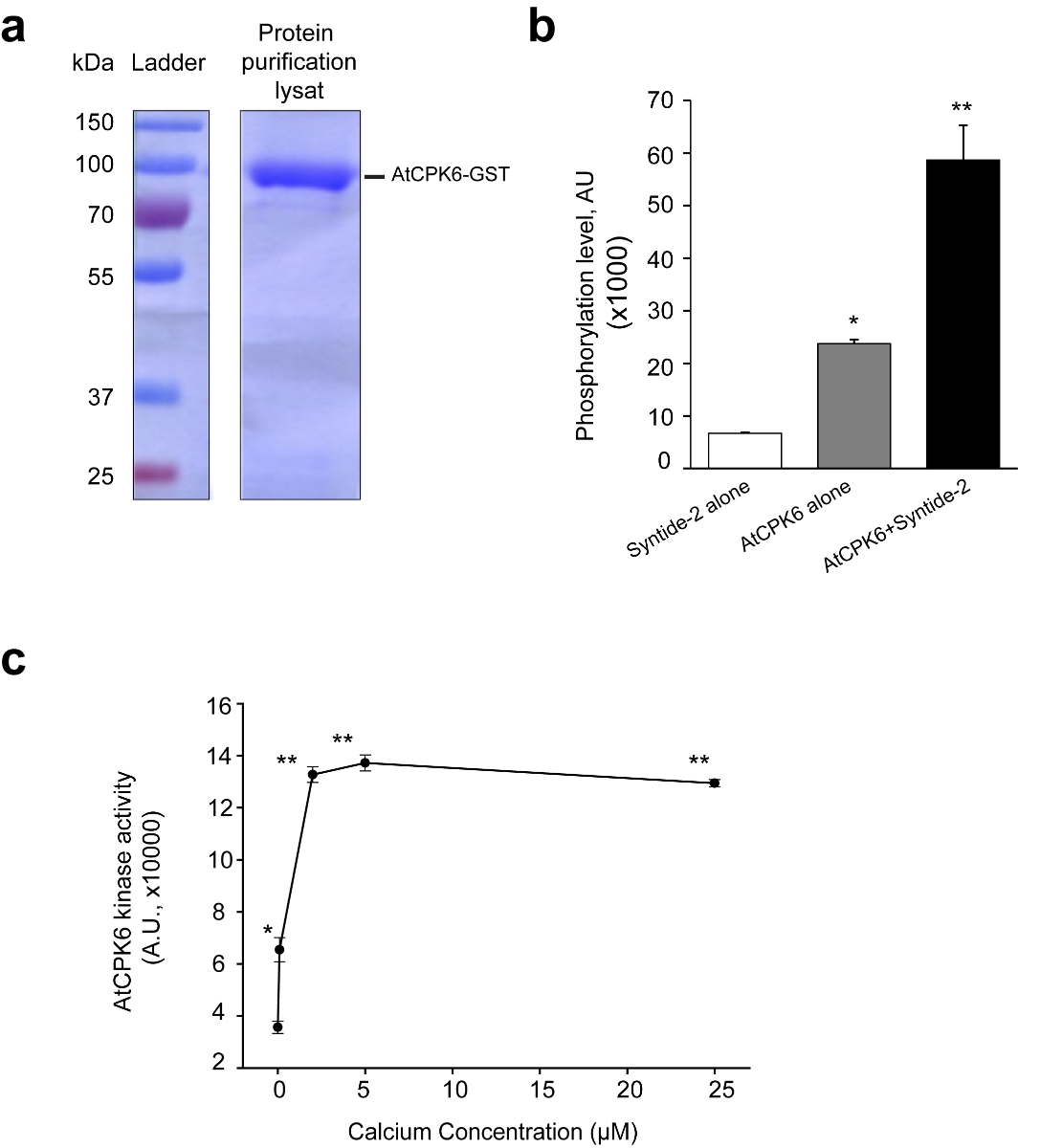


**Supplemental Figure S1:** Calcium-dependent activity of AtCPK6 recombinant protein. Representative gel SDS-page after Coomassie staining (a) showing a band for AtCPK6-GST at 80kDa after production and purification of the recombinant protein. Quantification of the activity of recombinant AtCPK6 kinase (b) using *in vitro* phosphorylation and syntide-2 peptide, n=3 independent experiments. AtCPK6 kinase activity (c) presented as function of calcium concentration (0.1 to 25µM), n=3 independent experiments.

Supplemental Figure S2


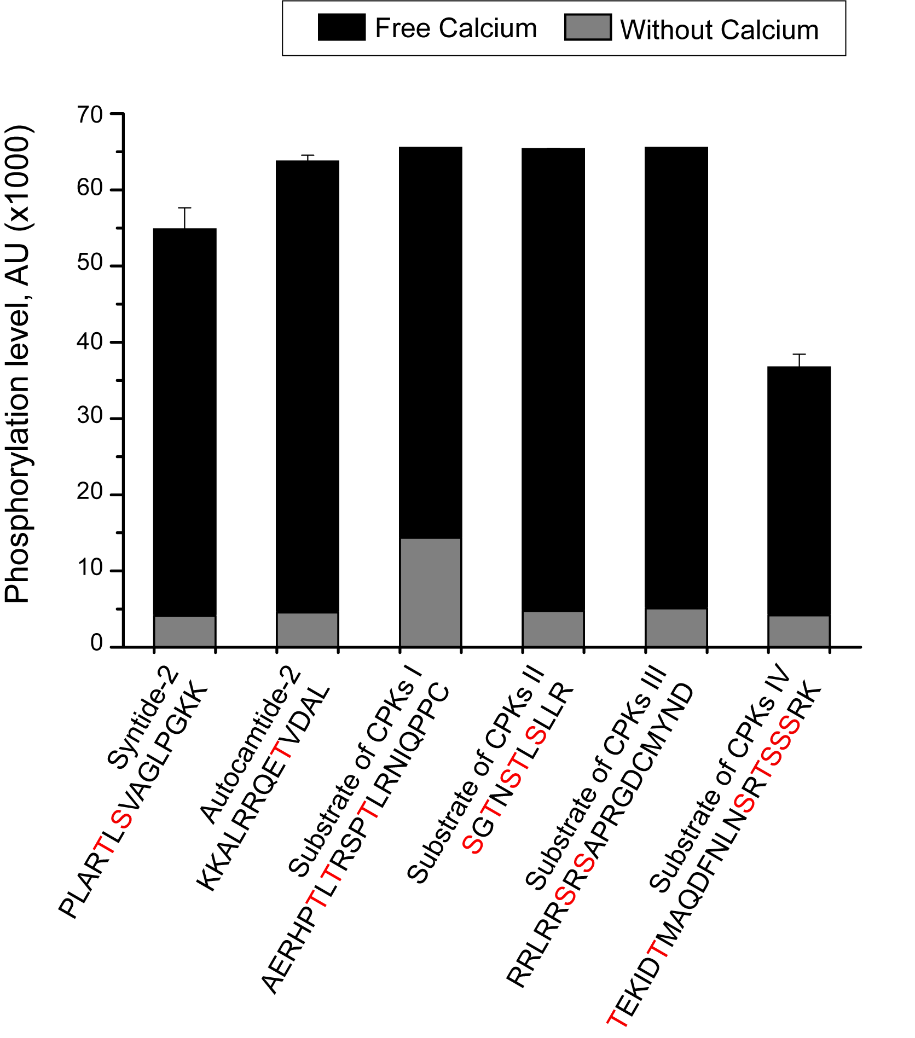


**Supplemental Figure S2:** *In vitro* phosphorylation of control peptides by recombinant AtCPK6. Quantification of *in vitro* phosphorylation of control peptides spotted on peptide array by recombinant AtCPK6 in presence (black bars) or absence (grey bars) of calcium. Results were obtained from one peptide array containing peptides in duplicates.

Supplemental Figure S3

**
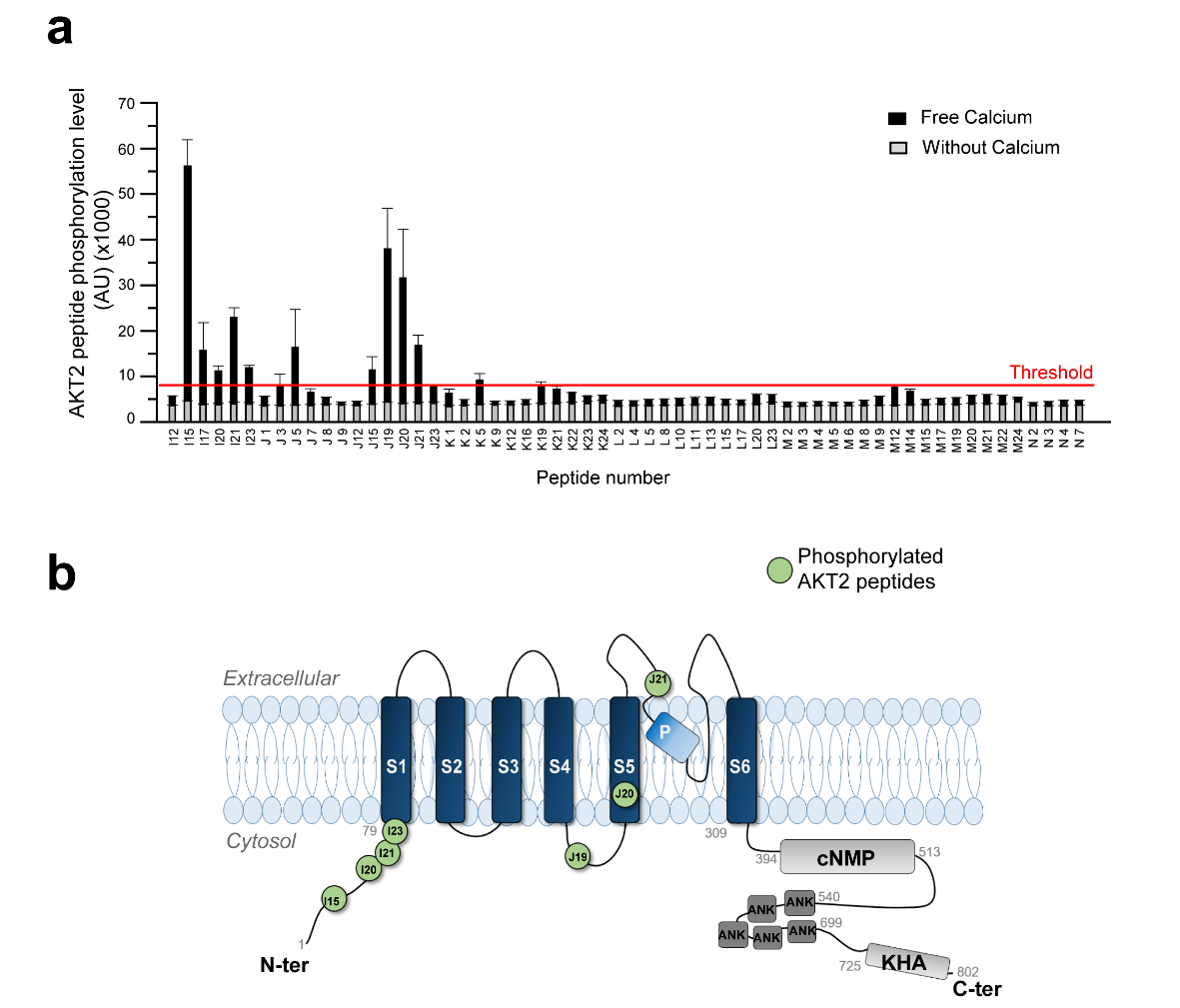
**

**Supplemental Figure S3:** Putative phosphorylation sites of AKT2 Shaker channel targeted by AtCPK6. Quantification of AKT2 peptides phosphorylation by AtCPK6 (a) in presence (black bars) or in absence of Ca^2+^ (grey bars). Results were obtained from one peptide array containing peptide in duplicates. Schematic drawing of the secondary structure of the AKT2 shaker channel subunit, highlighting the putative sites phosphorylated by AtCPK6 (green dots) as deduced from the peptide array. Ext, stand for extracellular, S1 to S6, P, cNMP, ANK and KHA stand for transmembrane segments 1 to 6, pore domain, putative cyclic nucleotide-binding domain, Ankyrin, and domain rich in hydrophobic and acidic residues, respectively.

Supplemental Figure S4

**
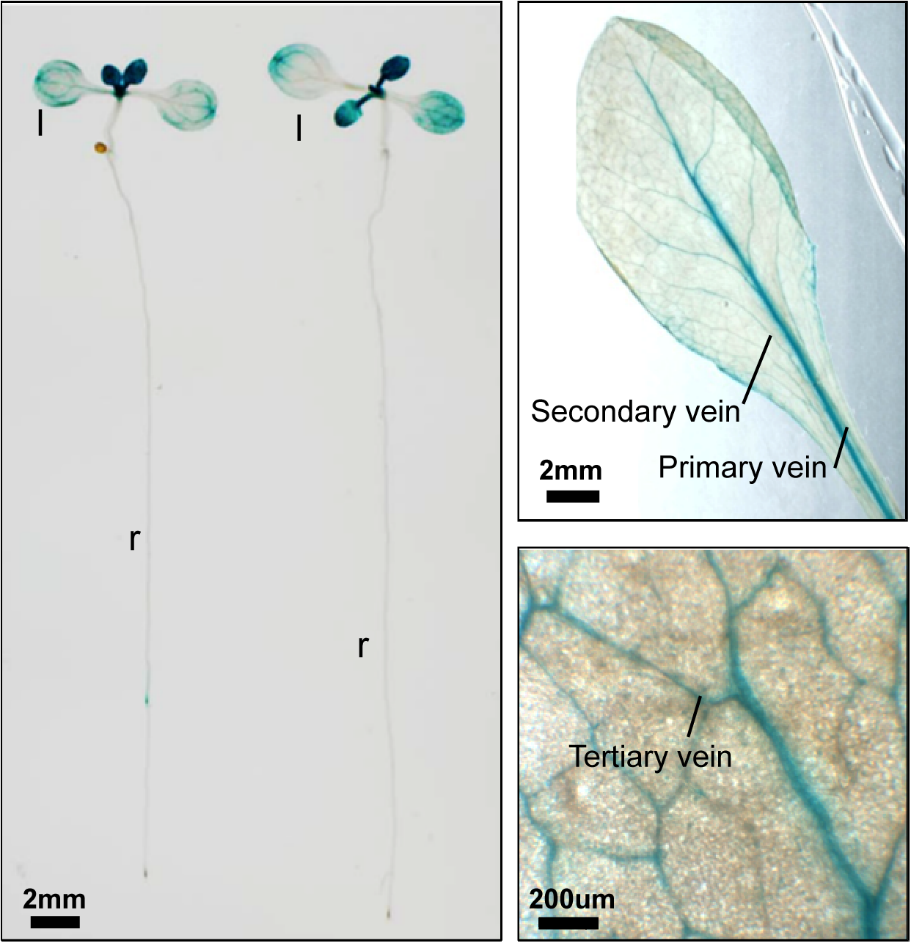
**

**Supplemental Figure S4: *KAT2* Expression pattern in *Arabidopsis thaliana.*** Representative pictures of p*KAT2*-GUS *Arabidopsis thaliana* after GUS staining (l, leaf; r, root).

**SUPPLEMENTAL TABLES**

**Supplemental table S1:** List of KAT2 peptides included in the peptide array

**
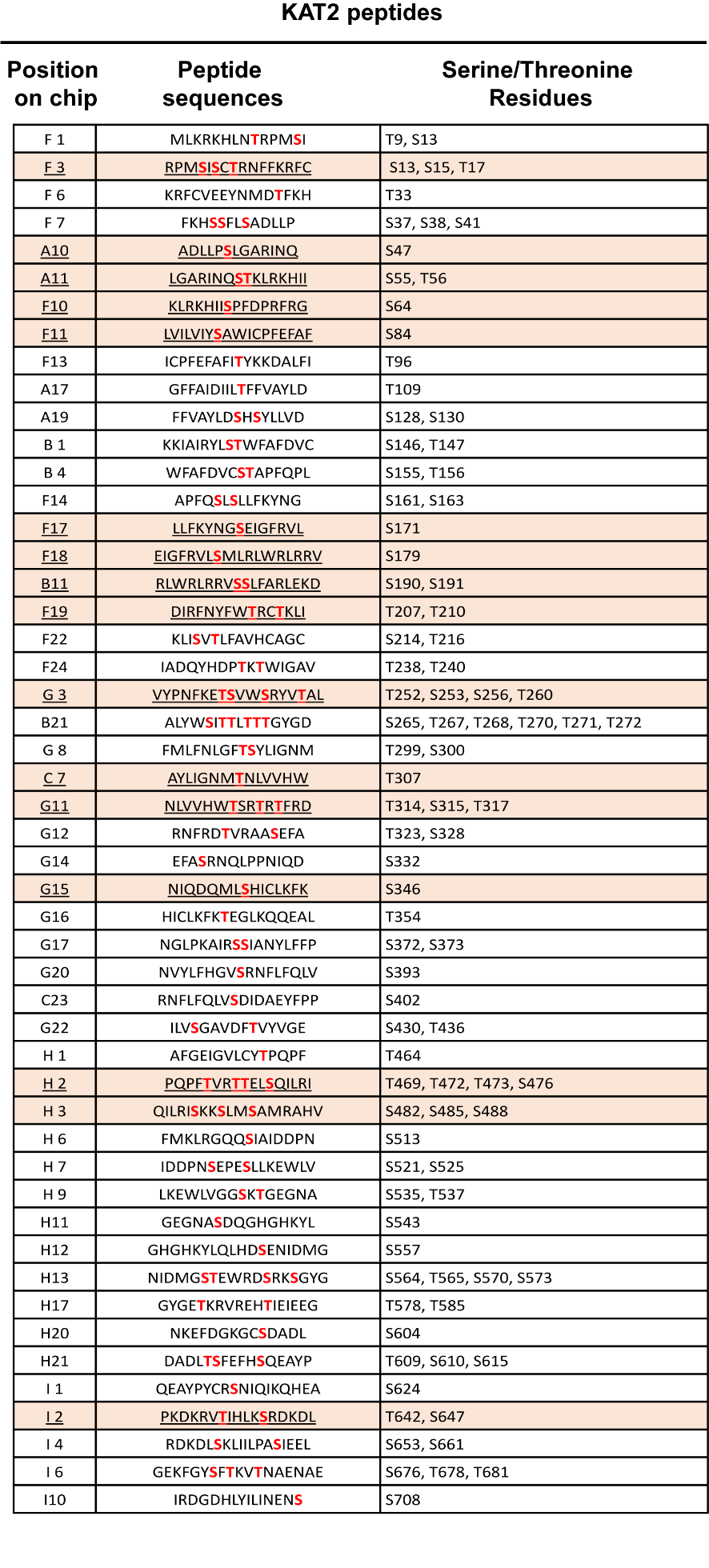
**

**Supplemental table S2:** List of AKT2 peptides included in the peptide array

**
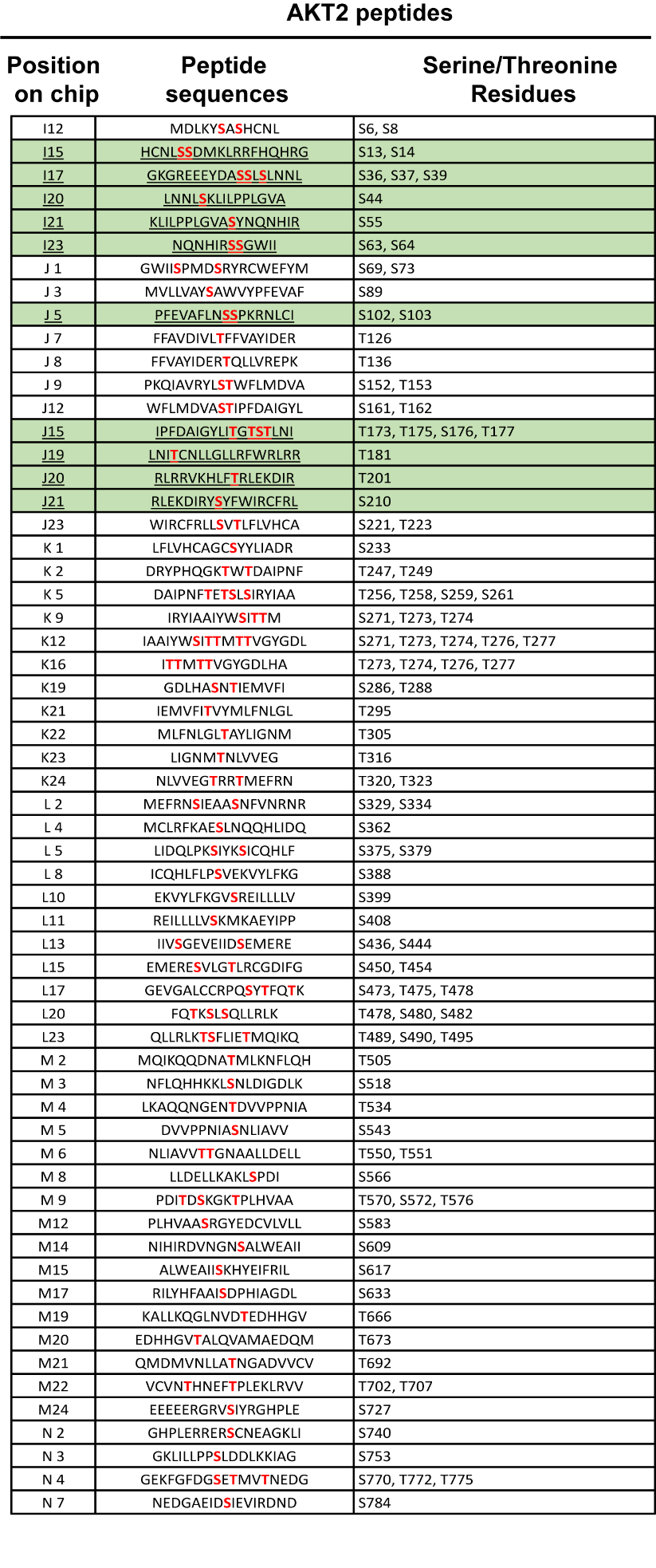
**

**Supplemental table S3:** List of primers used in this study for cloning

| **Primer name** | **Sequences** |
| --- | --- |
| *AtCPK6* FW3 | 5’-AGACAACGATGGACGGATTG-3’ |
| *AtCPK6* RV3 | 5’-CTCCCTACACCAGCATTTCC-3’ |
| *AtCPK6* promoter FW | 5’-GGAGATAGAACCAGTTTGAGCAACTGTGAGCTTCGC-3’ |
| *AtCPK6* promoter RV | 5’-TCCACCTCCGGATCCGAAACAAACTCTCTCTATTTCAC-3’ |
| *KAT2* FW | 5’-TCCTAGTCTCAGGGGCAGTGGA-3’ |
| *KAT2* RV | 5’-TGCATCGCCAACAACCGCCT-3’ |
| *AKT2* FW | 5’-AGGATCACCATGGCGTCACAGC-3’ |
| *AKT2* RV | 5’-TCTGCACCGTTCGTCGCCAG-3’ |
