# Supplementary figures and images for: Ca2+-Dependent Protein Kinase 6 Enhances KAT2 Shaker Channel Activity in Arabidopsis thaliana
Source: Int J Mol Sci. 2021 Feb 5;22(4):1596. doi: 10.3390/ijms22041596 (PMC7914964; doi:10.3390/ijms22041596)

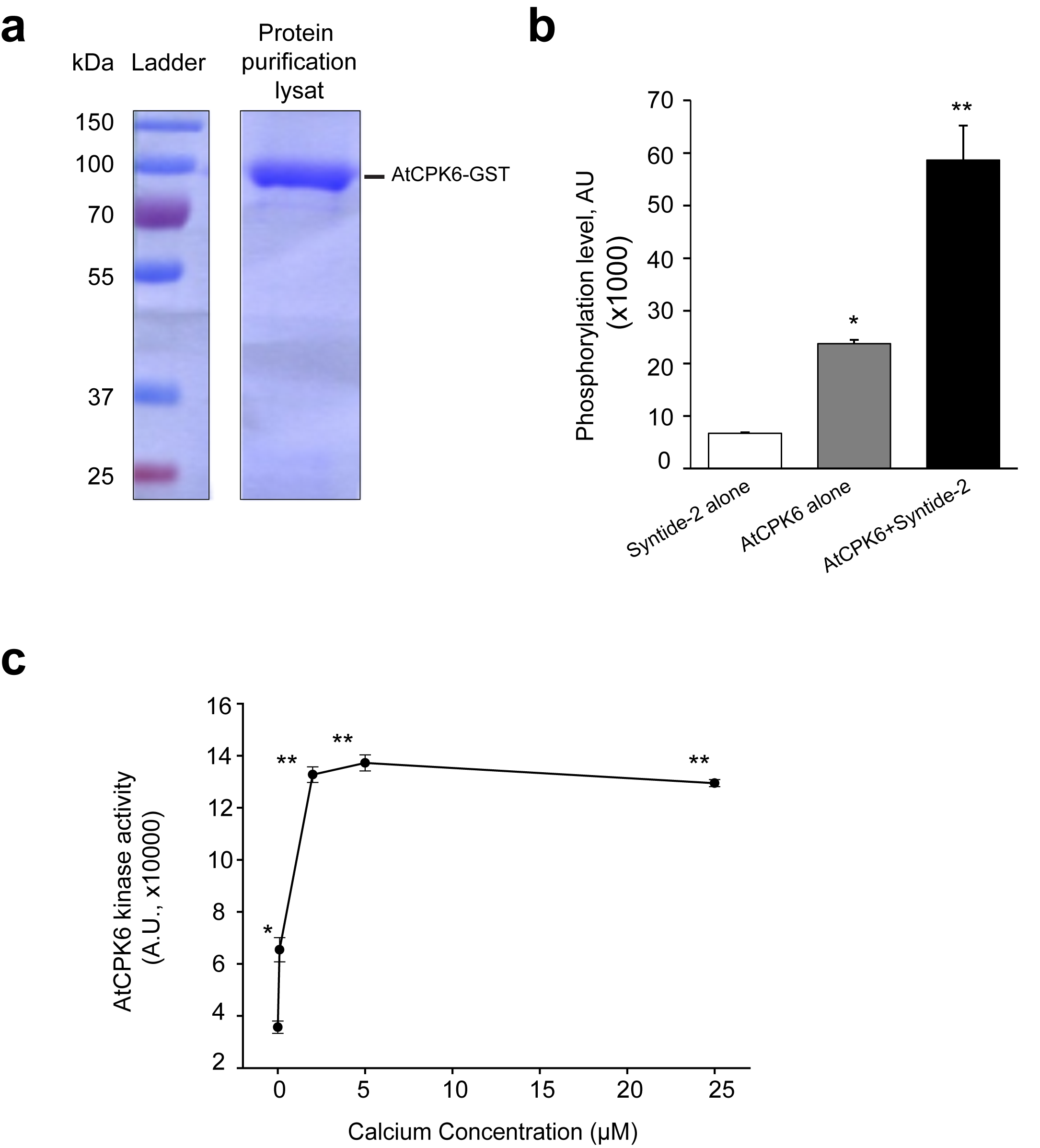

Supplement: Supplementary file 1 [file ijms-22-01596-s001.zip › Supp figure S1.tif]

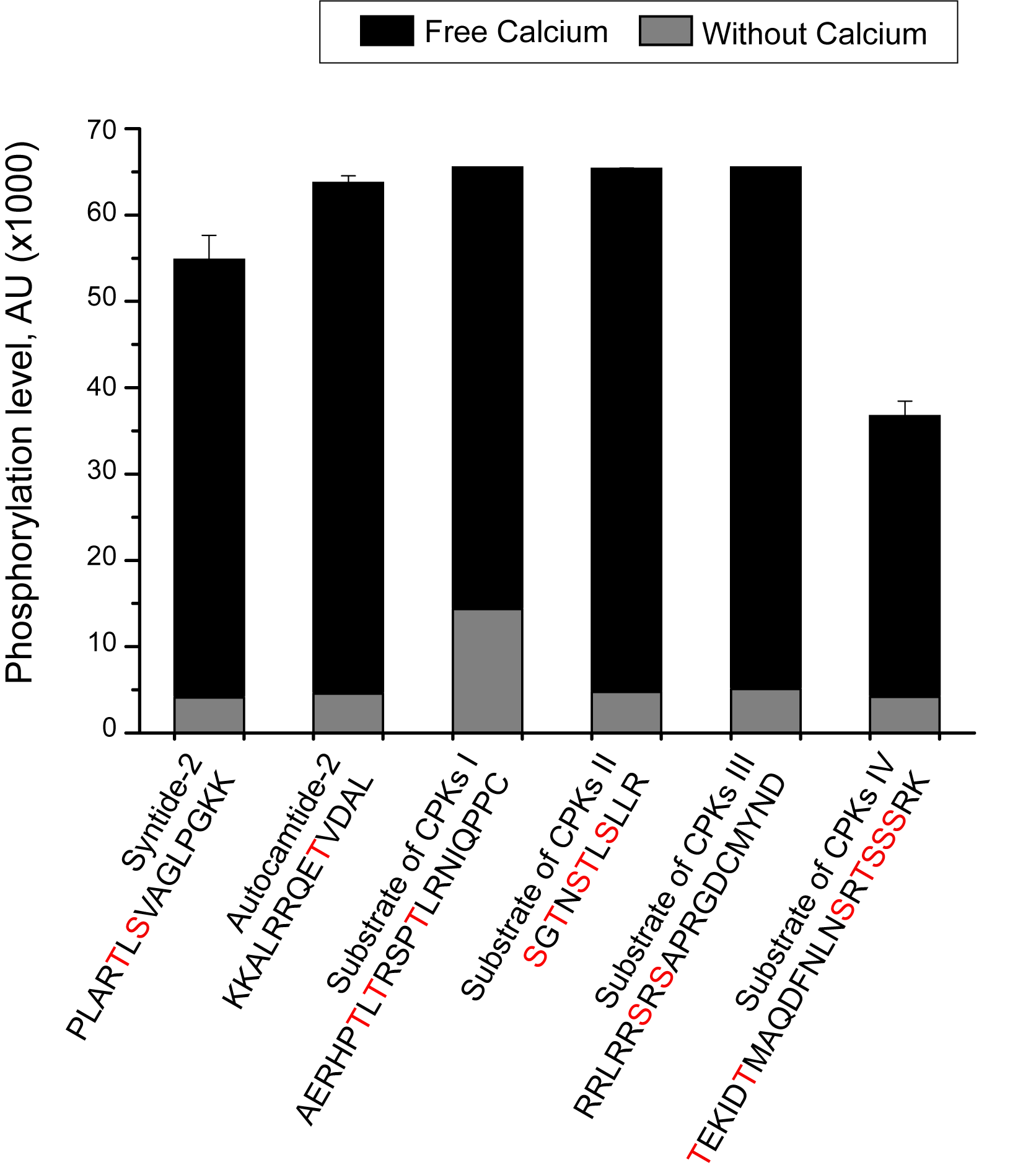

Supplement: Supplementary file 1 [file ijms-22-01596-s001.zip › Supp figure S2.tif]

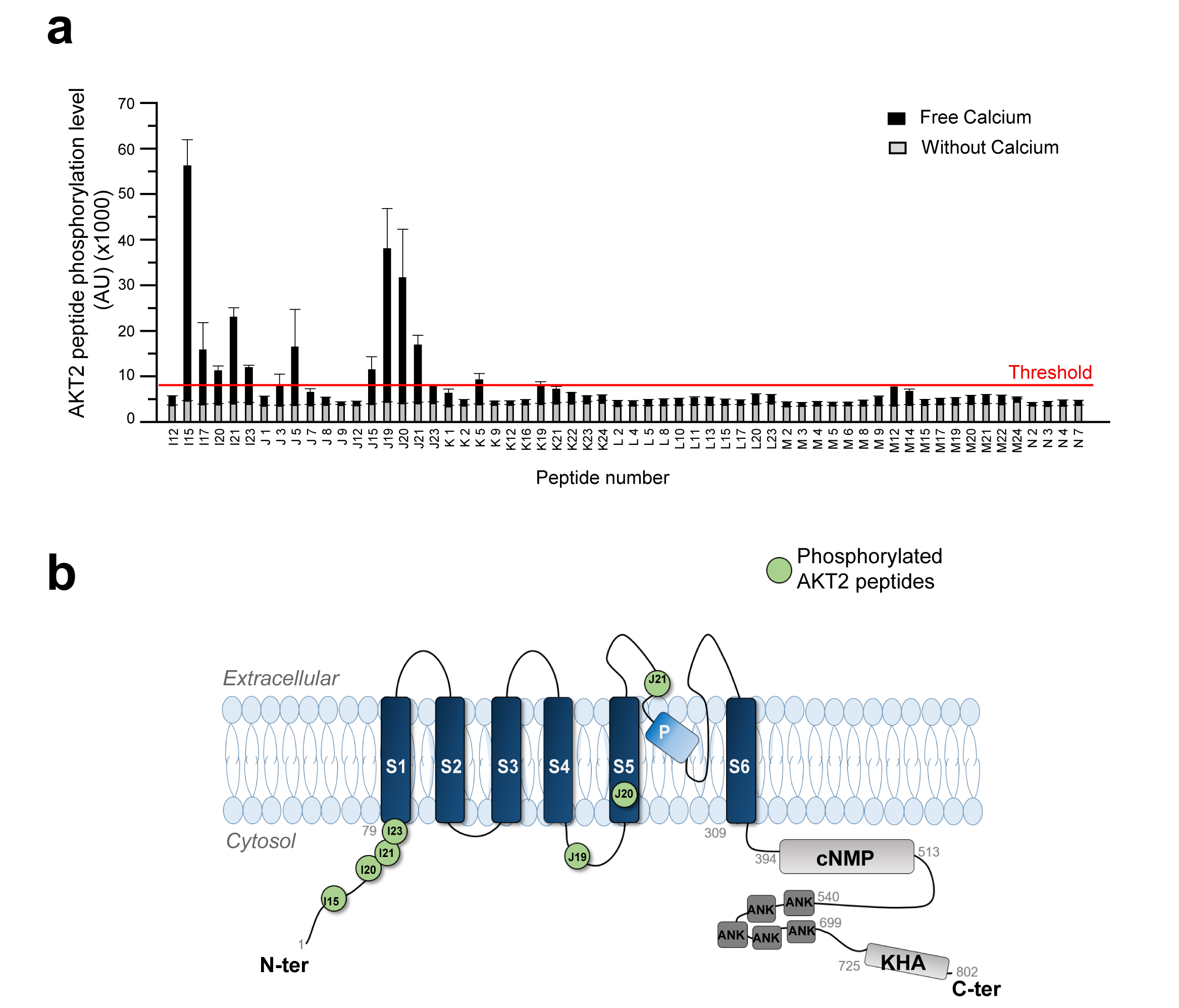

Supplement: Supplementary file 1 [file ijms-22-01596-s001.zip › Supp figure S3.tif]

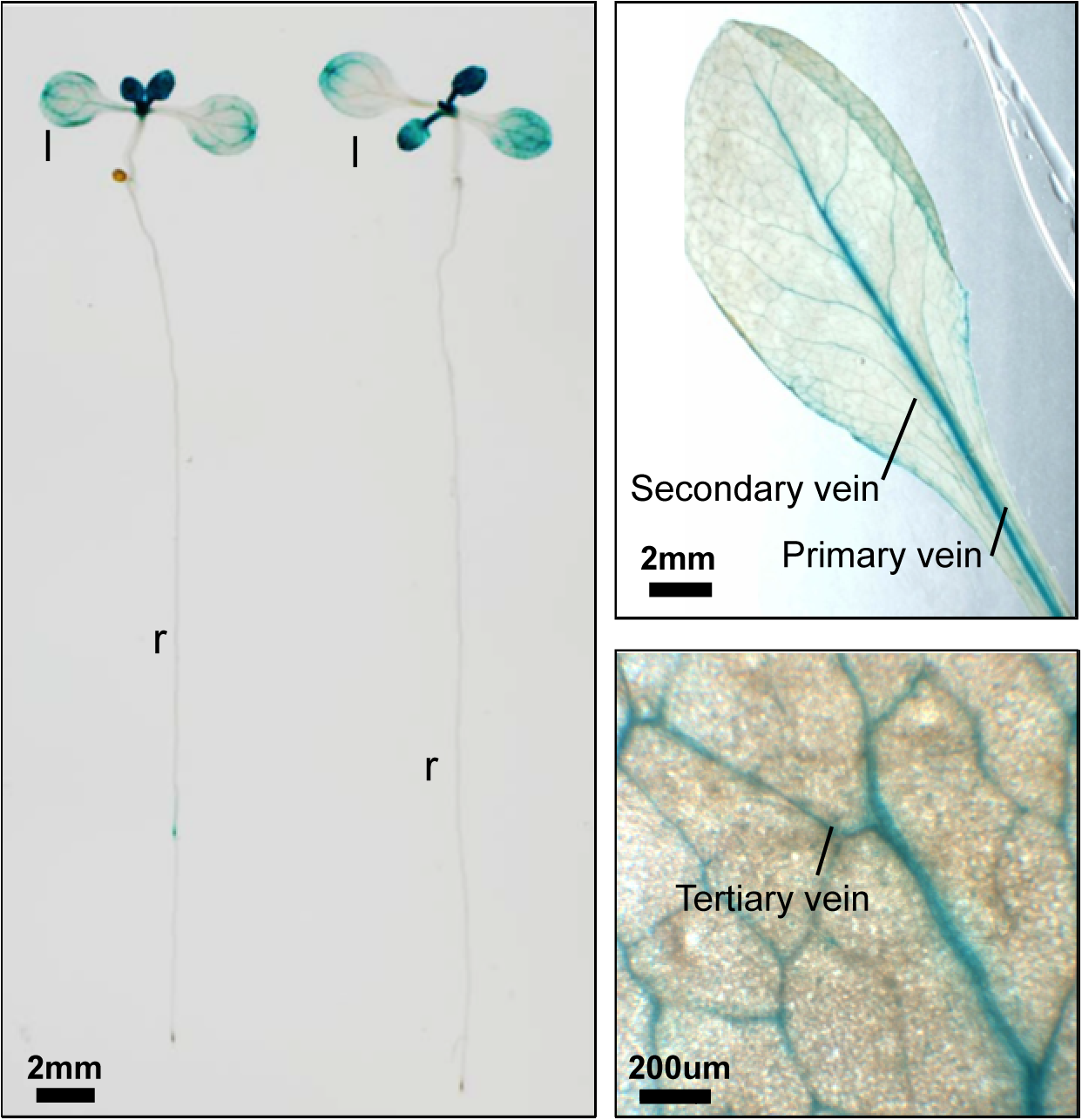

Supplement: Supplementary file 1 [file ijms-22-01596-s001.zip › Supp figure S4.tif]
